# Floral traits are associated with the quality but not quantity of heterospecific stigmatic pollen loads

**DOI:** 10.1186/s12898-020-00323-5

**Published:** 2020-10-06

**Authors:** Manon A. Peuker, Hannah Burger, Sabrina Krausch, Ulrich Neumüller, Manfred Ayasse, Jonas Kuppler

**Affiliations:** Institute of Evolutionary Ecology and Conservation Genomics, Ulm University, Albert-Einstein-Allee 11, 89081 Ulm, Germany

**Keywords:** Pollination, Community, Pollen transfer, Functional trait

## Abstract

**Background:**

In flowering communities, plant species commonly share pollinators and therefore plant individuals receive heterospecific pollen (HP). However, the patterns of HP transfers can deviate from patterns of plant-pollinator visitations. Although flower-visitor interactions are known to be mediated by floral traits, e.g. floral size or nectar tube depth, the explanatory power of these traits for HP transfer patterns remains elusive. Here, we have explored pollen transfer patterns at three sites in Southern Germany on three dates (early, mid and late summer). At the plant level, we tested whether flower abundance and floral traits are correlated with HP reception and donation. At the community level, we determined whether flower and bee diversity are correlated with network modularity and whether floral traits explain the module affiliation of plant species. We collected the stigmas of flowering plant species, analysed HP and conspecific pollen (CP) loads and measured floral traits, flower and bee diversity.

**Results:**

Our results show that the degree and intensity of HP reception or donation at the plant level do not correlate with floral traits, whereas at the community level, the module affiliation of who is sharing pollen with whom is well-explained by floral traits. Additionally, variation in network modularity between communities is better explained by plant diversity and abundance than by bee diversity and abundance.

**Conclusions:**

Overall, our results indicate that floral traits that are known to mediate flower-visitor interactions can improve our understanding of qualitative HP transfer but only provide limited information about the quantity of HP transfer, which more probably depends on other floral traits, flower-visitor identity or community properties.

## Background

In flowering communities, heterospecific pollen (HP) transfer is common and can result in negative consequences for plant reproductive success such as reduced pollen tube growth and seed production [1–6]. HP transfer mainly occurs when various plant species share the same flower visitors [7], although wind-dispersed pollen and other random events may also play a role [3, 8]. During foraging, insects often visit and collect pollen from flowers from multiple plant species and may consequently transfer con- and heterospecific pollen (CP / HP) to a stigma [9]. However, the CP-/HP loads on stigmas often do not match flower-visitation patterns [7, 10, 11].

Within a community, flower-visitor interactions can be summarized in networks depicting multi-species interactions [12, 13]. Such networks can also depict interactions based on pollen transfer instead of flower visitors. If flower-visiting insects are the main vector for pollen transport between plant individuals, factors that mediate flower-visitor interactions might also mediate pollen transfer patterns. Common mediators are: flower abundance or diversity, flower visitor diversity or floral traits [14, 15].

Abundant plant species often receive more visits from a more diverse insect community [14, 16]. Therefore, individuals of abundant plant species might receive more CP/HP or their pollen might be distributed across a larger number of diverse plant species. Whereas this relationship between plant abundance and pollen transfer has been observed in nature [17], floral traits or pollination mode can contribute equally or more to pollen transfers [4].

In diverse plant and animal communities, increasing competition between flower visitors can lead to increasing specialization and resource partitioning [15, 16, 18]. As a consequence, flowering communities with a high species richness are often separated into different sub-networks of interacting species, i.e. are more modular [19, 20]. Within one module, plant species share the same flower visitors thereby increasing the chance of exchanging pollen with each other. Thus, in pollen transfer networks, modularity may increase with plant and flower-visitor diversity.

Floral traits, such as morphology, colour or scent, have been shown to mediate flower-visitor interactions [15, 21–24]. During foraging, flower visitors of different functional groups or species often prefer plant species with specific floral phenotypes [25]. Thus, plant species displaying the expression of similar floral traits might have a high likelihood of receiving pollen from or of donating pollen to each other. For example, the radius of the floral tube can partially explain the modularity in pollen transfer networks in terms of the presence or absence of interactions, i.e. its quality [7]. However, patterns for the intensity or quantity of pollen transfer, i.e. the amount of CP / HP pollen received or donated, are not well explained by floral traits [7, 26].

Associations between floral traits, HP reception or donation and network properties are variable and even contradictory [3, 26, 27]. In general, stigma size, flower symmetry and floral size increase the likelihood and intensity of HP reception [3, 27, 28]. Short styles have been suggested to increase or decrease HP susceptibility [1, 4, 27]. The picture is similar for network properties. The in-degree, i.e. the number of plant species that receive pollen, is positively correlated with style length [26, 27]. In contrast, the number of plant species that donate pollen, i.e. out-degree, is either not correlated [27] or the correlation patterns differ [26]. However, correlation studies concerning associations between pollen transfer, floral traits and community patterns are scarce [26].

The aim of our study has been to determine the way that floral traits, flower and bee abundance and diversity are associated with patterns of pollen reception and donation in natural communities. We have investigated whether these predictors are correlated with properties of pollen transfer networks. In order to analyse HP and CP loads, we have collected the stigmas of flowering plant species at three sites in Southern Germany on three different dates (early summer, mid-summer and late summer). Further, we have measured morphological floral traits (stamen length, inflorescence diameter, nectar tube width, nectar tube depth, floral display size and style length) and flower and bee abundance and diversity. Specifically, we have asked the following four questions: (I) Are pollen receipt and donation (in- and out-degree) and its intensity (weighted in- and out-degree) correlated with specific floral trait expression or flower abundance? (II) Is the modularity of pollen transfer networks linked to community properties such as bee or plant species richness? (III) Can floral trait expressions explain species module affiliation in pollen transfer networks? (IV) Do plant species with similar floral trait expression receive a similar HP percentage?

## Results

In total, 116,954 pollen grains (110,365 CP and 6,589 HP) on 1,117 stigmas resulting in 347 interspecific pollen transfers (IPT) were sampled across all communities (Table 1). Between communities, the number of IPTs ranged from 8 to 91. The number of HP grains per stigma was generally small compared with the number of pollen grains (mean %-CP load per stigma ± sd, 86.98 ± 26.75%, range 0–100%). In general, CP was present on ~ 99% (*n* = 105), HP on ~ 42% (*n* = 470) and no pollen only on ~ 0.05% (*n* = 55) of all stigmas. The number of average pollen grains per stigma ranged from 1.3 to 748.8. The maximum number of HP grains found on one stigma was 386 (*Campanula rotundifolia*) and the maximum number of pollen grains of different plant species identified on one stigma was 6 (*Campanula rotundifolia*, *Centaurea jacea*, Asparagaceae sp.2).

**Table 1:** Descriptive results of sampling and pollen transfer within all nine communities

	EB I	EB II	EB lII	HT I	HT II	HT III	RB I	RB II	RB III
No. Species*	4 (4)	17 (20)	7 (9)	7 (9)	16 (19)	17 (19)	8 (9)	16 (20)	22 (24)
Total no. stigmas	48	160	46	72	178	156	71	162	224
Total no. pollen grains	11,030	21,564	8373	8409	13,956	11,279	9446	9324	23,573
Total no. CP grains	10,472	20,712	8187	8056	12,211	10,883	9280	8668	21,896
Total no. HP grains	558	852	186	353	1745	396	166	656	1677
Total no. links^+^	8	48	14	12	57	57	11	49	91
%Donor	25	43.75	28.57	42.86	20	47.06	37.5	37.5	36.36
%Receptor	50	56.25	57.14	57.14	73.33	47.06	25	56.25	54.54
%Donor-Receptor	25	0	14.29	0	6.77	5.88	37.5	6.25	9.1
Corr. In- and out-degree	− 0.30^NS^	− 0.23^NS^	0.62^NS^	− 0.11^NS^	− 0.23^NS^	− 0.09^NS^	− 0.53^NS^	− 10.19^NS^	− 0.10^NS^
Modularity	0	0.38	0.1	0.25	0.47	0.48	0.31	0.56	0.47

EB = Eselburger Tal, HT = Hirschtal, RB = Reichenbach. Roman numerals show sampling date: I = early summer, II = mid-summer, III = late summer. No. species = Total number of species, Total no. stigmas = Total number of collected stigma, Total no. pollen grains = Total number of pollen grains found on all stigmas, Total no. CP grains = Total number of CP grains found on all stigmas, Total no. HP grains = Total number of HP grains found on all stigmas, Total no. links = Total number of links found in the plant-plant pollen transfer network, %Donor = Percentage hub-donor species in the network, i.e. species that donated HP to more species than from which they received HP, %Receptor = Percentage receptor species in the network, i.e. species that received HP from more species than to which they donate HP(??), %Donor-Receptor = Percentage of species that donated and received pollen from the same number of species. Corr. In- and out-degree = Pearson product correlation *r* for in- and out-degree for each species within a community; *ns* non-significant. Modularity = Modularity for HP transfer network calculated using the optimization algorithm of Blondel et al. [43]

*No. species = Number of species from which stigmas were collected (Number of flowering plant species. Some species were under protection or only present with < 3 individuals and were excluded)

^+^Total no. links = in each network, including links resulting from species that were not on our plots

**Fig. 1:**
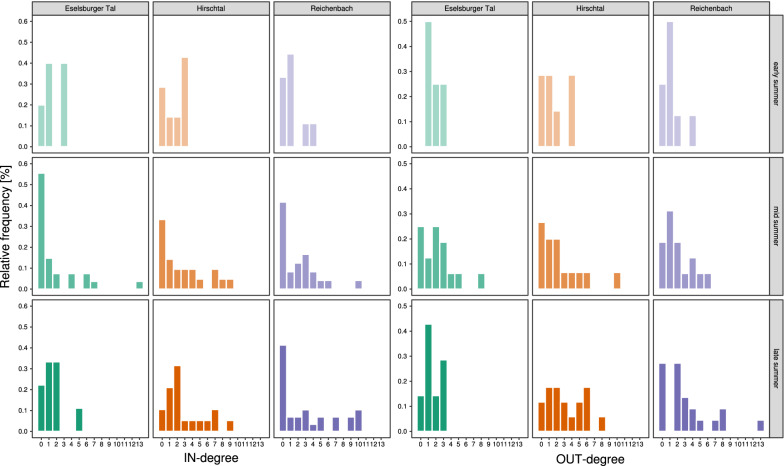
Frequency distribution of the in- and out-degree values of each species in pollen transfer plant-plant networks. Different colours represent different locations: Eselsburger Tal (green), Hirschtal (orange), Reichenbach (purple). Different colour alpha values (i.e. shading) represent sampling date: early summer (0.4), mid-summer (0.7), late summer (1). The in-degree is the number of incoming links, i.e. number of species of which HP grains were found on the stigma. The out-degree is the number of outgoing links, i.e. the number of species to which HP grains were donated

The frequency distribution of in- and out-degree was right-skewed and variable in all IPT networks (Fig. 1). For the in-degree, several zeros occurred attributable to HP grains of plants that were currently not flowering on our plots but that might have been flowering in the surroundings or some time before the sampling was conducted. In all IPT networks, most of the species (range: 75 – 100%) received HP grains from at least one species. Approximately 46% of these species received pollen from one or two other species, whereas some species received pollen from a large number of species. For example, the highest in-degree (= 13) was found for *Campanula rotundifolia* (Eselsburger Tal, mid-summer), whereas an in-degree of 10 was recorded for four other species: *Agrimonia eupatoria* (Reichenbach, mid summer), *Scabiosa columbaria*, *Thymus pulegioides* and *Heliantemum nummularium* (Reichenbach, late summer). The patterns were similar for the out-degree case but, on average, species had a higher in- than out-degree value. In all IPT networks, most of the species (range: 71.4–100%) donated HP grains to at least one species. Approximately 62% of these species donated pollen to one or two other species. The highest out-degree (= 13) was found for *Daucus carota* (Reichenbach, late summer), whereas an in-degree of 10 was found for *Lotus corniculatus* (Hirschtal, mid-summer). The percentage of species that acted as hub-donor or -receptor species varied between communities but, in general, more species were identified as hub-donors than receptors (Table 1). Therefore, no statistically clear correlation was detected between the in- and out-degree values of species in all nine communities (Table 1).

Most floral traits showed no statistically clear correlation with either the in- and out-degree (Additional file 1-1). The only statistically clear associations were a negative correlation of the out-degree with stamen length (*z* = − 2.031, *p* = 0.042) and a positive correlation of the out-degree with plant abundance (*z* = 2.480, *p* = 0.013).

Similar patterns were found for the weighted in- and out-degree values (Additional file 1-2). Weighted in-degree showed a statistically clear correlation with nectar tube width (*z* = 2.004, *p* = 0.045) and floral abundance (*z* = 2.344, *p* = 0.019) and a positive trend with floral display (*z* = 1.907, *p* = 0.057). Weighted out-degree showed only a statistically clear correlation with floral abundance (*z* = 5.535, *p* < 0.001) and a negative trend for stamen length (*z* = -1.859, *p* = 0.063) and nectar tube width (*z* = 2.930, *p* = 0.087).

**Fig. 2:**
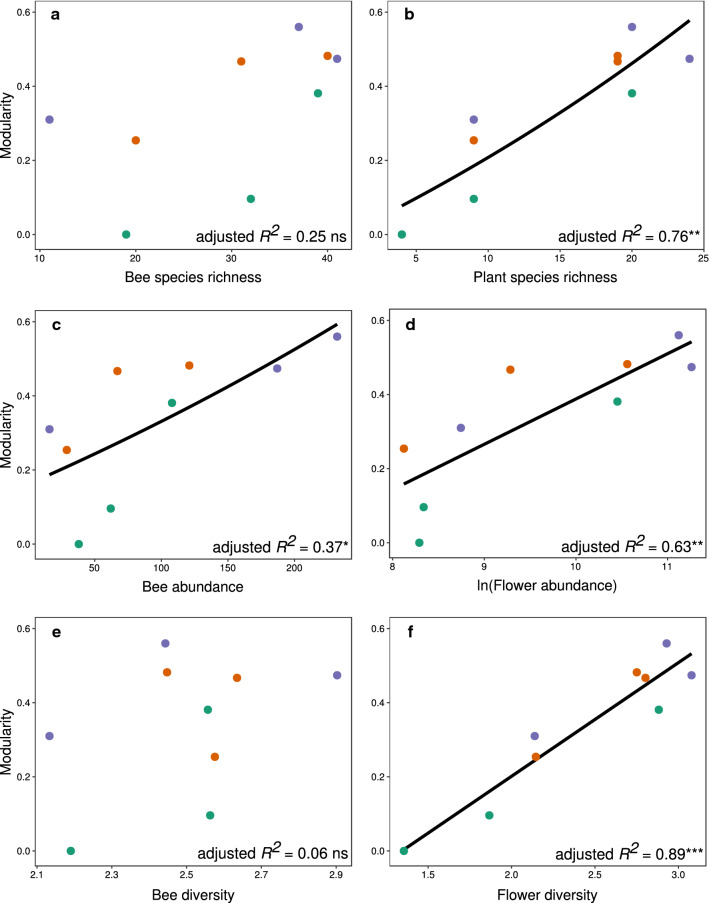
Correlation between the modularity of the pollen transfer plant-plant network and community parameters. Community parameters are: (**a**) bee species richness, (**b**) bee abundance, (**c**) bee diversity, (**d**) plant species richness, (**e**) flower abundance (ln-transformed) and (**f**) flower diversity. Diversity was measured as the Shannon–Wiener index. Different colours represent different locations: Eselsburger Tal (green), Hirschtal (orange), Reichenbach (purple). Each location was sampled once in early, mid and late summer. Black lines are lines of best fit derived from linear models. Adjusted *R*^*2*^ are given in the lower right-hand corner of each graph (full results shown in Additional file 1-4). ns = non-significant, *p < 0.05, **p < 0.01, ***p < 0.001

Plant diversity explained more variation in modularity than bee diversity across the nine communities (Fig. 2, Additional file 1-3). The effect of bee species richness (adjusted *R*^*2*^ = 0.25^ ns^) and bee diversity (adjusted *R*^*2*^ = 0.06^ ns^) on modularity was statistically unclear, whereas for bee abundance, we found a statistically clear relationship with modularity (adjusted *R*^*2*^ = 0.37*). Modularity showed a statistically clear increase with plant species richness and with flower abundance and diversity of each community (adjusted *R*^*2*^*;* 0.76** (species richness); 0.63** (flower abundance); 0.89*** (flower diversity). All three factors were correlated (Pearson's product-moment correlation: *r* > 0.86***).

**Fig. 3:**
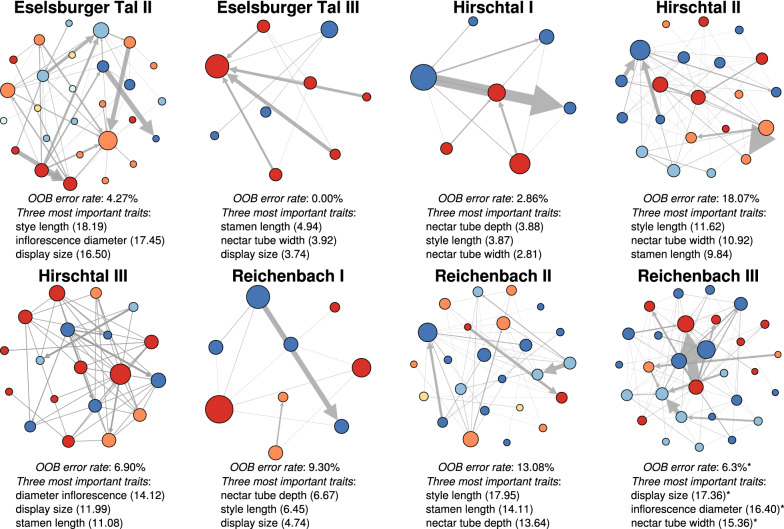
Plant-plant networks based on pollen transfer patterns of eight sampled grassland communities. Each node represents one plant species and each link represents HP transfer between two species. Direction of arrows indicates HP transfer from donor to receptor species and width of arrows indicates the number of transferred pollen grains. Nodes with the same colour are in the same module within one community. Numbers in community names show sampling date: I = early summer, II = mid-summer, III = late summer. Eselsburger Tal I is not shown as no modules were detected. OOB: the estimate of error rate from random forest analysis shows the percentage of misclassifications, i.e. assignment to the wrong module. The three most important variables for the module classification are given for each community (Table 2). The full results of the random forest analysis are given in Additional file 1-5. For RB III, two solutions of the modularity algorithm (Blondel et al. 2008) are shown as both were equally likely: solution one with four modules / solution two with five modules because one module was split into two plant-plant networks (computed in *Gephi* version 0.9.2 [41] by using the Fruchterman Reingold layout)

Additionally, floral traits were good predictors for the module composition (randomForest OOB estimate of error rate, mean ± sd: 7.60 ± 5.81%, Fig. 3, Table 2, Additional file 1-4). However, those floral traits that had the highest importance for module separation differed between communities (Table 2, Fig. 3). Display size and style length were among the two most important traits for module separation in five out of eight communities. The other traits were among the most important traits in three or four communities.

**Table 2:** Importance of measured floral traits for module separation based on random forest analysis

Floral trait	EB II	EB III	HT I	HT II	HT III	RB I	RB II	RB III
Stamen length	13.76	4.94	1.58	9.84	11.08	2.94	14.11	12.01/13.77
Inflor. diameter	17.45	2.52	2.40	7.53	14.12	2.87	10.86	16.40/18.35
Nectar tube depth	10.38	1.27	3.88	8.01	7.11	6.67	13.64	11.88/13.01
Nectar tube width	13.28	3.93	2.81	10.93	5.06	3.12	9.11	15.36/15.66
Display size	16.50	3.73	2.12	9.32	11.99	4.74	13.63	17.36/18.36
Style length	18.19	2.29	3.87	11.62	8.96	6.45	17.94	12.83/15.96

Larger numbers indicate greater importance for module classification. Inflor. diameter = Inflorescence diameter. Location: Eselsburger Tal (EB), Hirschtal (HT), Reichenbach (RB). Sampling date: early summer (I), mid-summer (II), late summer (III). For RB III, two solutions of the modularity algorithm [43] are shown as both were equally likely: solution one with four modules / solution two with five modules because one module was split into two. Eselsburger Tal I is not shown as no modules were detected

The percentage of received HP percentage showed no statistically clear correlation with floral traits (Additional file 1-5). However, a statistically clear positive relationship was found with floral abundance (${\mathrm{\rm X}}_{1}^{2}$ = 57.99, *p* < 0.001) but the explanatory power was low (*R*^*2*^_*marginal*_ = 0.01, *R*^*2*^_*conditional*_ = 0.65).

## Discussion

In contrast to flower-visitor interaction networks [22, 25, 29], the outcome of these interactions, namely pollen transfer, and its association with floral traits has only been investigated in a few communities [17, 26, 27]. Our results show that modularity is well-explained by floral traits but not the quantity and intensity of HP reception or donation. Additionally, network structure is correlated with plant diversity, whereas bee diversity is indicated as being less important. Therefore, we suggest that the topological structure in pollen transfer networks can be explained by the measured floral traits, whereas the intensity or degree of HP transfer is mediated by other factors potentially including various floral traits.

HP receipt and donation (i.e. in- and out-degree) and its intensity (i.e. weighted in- and out-degree) were poorly explained by most of the floral traits. Only the size of the flower entrance (nectar tube width) was positively correlated with weighted in- and out-degree and stamen length negatively with (weighted) out-degree. This indicates that flowers that are easier accessible have a higher quantity of HP and that plant species with short and more hidden stamens, can donate HP to more species [26]. All our communities show an abundance of bees and, particularly, bumble bees, which regularly visit complex flowers, e.g. Lamiaceae, or flowers with deep nectar tubes that are not accessible to many other floral visitors [22]. These plants might have received a large number of visits by bees resulting in an increased out-degree value. This potential effect of abundance and flower-visitor community is in congruence with previous results that have indicated non-consistent out-degrees between communities [30]. Additionally, floral abundance had a positive effect on weighted in-degree and (weighted) out-degree numbers. Therefore, we suggest that, in communities dominated by generalist bees (in our case only ~ 10–20% specialist species), pollen donation is a function of floral abundance or flower-visitor community rather than of species-specific floral traits.

The modularity of the sampled communities was better predicted by flower abundance and diversity than by those of bees. An increasing flower diversity often results in a higher specialization and higher resource partitioning of pollinator species [16]. This higher specialization limits the number of visited plants and thus might limit the HP transfer between multiple species. Further, in the sampled communities, hyper-generalized honeybees, whose presence increases the interconnectedness of plant species [26], are not ubiquitously found [pers. observ.]. Additionally, the low explanatory power of bee diversity for modularity indicates that other flower-visitor species such as syrphid flies need to be included in the measurements. For bees, only the factor abundance is positively correlated with modularity. Therefore, we suggest that, in our communities, the number of bees rather than the species richness increases resource partitioning and, thus, potentially increases modularity.

We have found that module affiliation is well-explained by floral traits. This indicates that plant species that share the same visitors receive and donate pollen from each other [27]. However, the intensity and quantity of pollen reception and donation is not well explained by measured traits. Therefore, we suggest that the measured traits explain the qualitative part of the interactions (interaction/no interaction) but not the quantitative part. Similarly, pollinator moves between plant species have also been shown to explain the plant species from which HP is received, but not the number of HP grains received [7].

Further, traits that are most important for the separation of plant modules differ between season and site. Even in communities with overlapping plant species, trait importance differs and, thus, none of our measured traits provides a general explanation for interspecific separation. Only display size and style length seem to be more important than other measured traits. The first is related to the width of the flower entrance and, therefore, the accessibility of pollen for the various flower visitors. The second indicates that style position affects HP transfer. Other traits, with lower importance, such as stamen length or nectar tube depth also indicate easier access of the flower visitor to pollen or might be an indicator of different floral morphology. These traits have also been found to be important for pollen transfer patterns in communities in Hawai’i and in high mountain areas [5, 27, 31]. Hence, we suggest that floral traits are important for attraction/efficiency but only some traits (and potentially different ones) are crucial for pollen transfer patterns, which might also depend on the composition of the flower-visitor community.

We found no correlation between floral traits and received HP reception but a positive correlation with floral abundance. This partly contradicts previous studies that have found no correlation with floral traits or that have shown that the %HP received is influenced by species properties such as floral symmetry, corolla openness and degree of style exsertion [1, 26, 27]. Additionally, the HP/CP ratio can vary between different species and years and thus suggests an impact of climatic factors [4]. We suggest that these differences between our findings and previous results can be explained by differences in the sampled communities. Whereas the results of Ashman and Arceo-Gómez [1] are based on a meta-analysis, Fang and Huang [27] and Fang et al. [4] have explored sub-alpine communities that differ in pollinator composition. This indicates that other factors in our planar and kollin meadow communities are more important than in other communities.

Additionally, the present associations between floral traits and HP reception might only become apparent when accounting for differences in flower-visitor interaction frequency. As the number of interactions and the identity of the flower visitors can affect the transported pollen, it can also influence HP reception [11, 32, 33]. However, flower-visitor interaction patterns do not necessarily match HP pollen transfers [7, 10, 11] and stigmatic pollen loads are consistent across years [4] despite the potentially large variation in interactions between years. These two points suggest that additional plant-based factors, such as floral traits, are important for determining stigmatic heterospecific pollen loads. Further, visitation frequency is often a poor proxy for efficient pollination, i.e. single pollen deposition [34]. Single visit pollen deposition and visitation frequency provide important information on the relative contribution of different taxa visiting a plant species but might be limited when focussing on broader community questions about pollination outcomes and patterns, stigmatic pollen load or seed set [35]. Therefore, the incorporation of interaction frequency and the pollen deposition of different animal species will provide an additional insight and a more mechanistic understanding for the general pollen transfer patterns found here.

As discussed above, floral traits are not or only partly correlated with the intensity or degree of HP transfer [1, 3]. Thus, we speculate that only a subset of floral traits such as stigma size or position influence the HP transfer/reception of plants. Fang et al. [4] have suggested that plants deal with HP either by tolerating it or by avoiding it. Therefore, we propose that HP reception also serves as a separate selection pressure for floral traits. To date, pollinator-mediated selection is usually separated into pollinator attraction and efficiency, whereby traits such as nectar tube depth are used in terms of pollinator efficiency [36]. For plants, traits relevant for pollinator attraction/efficiency and HP reception should presumably be separated, i.e. an adaption to one selection pressure might not influence the other, whereas some traits such as floral display size or stigma position may influence both pollinator attraction/efficiency and HP reception. Therefore, future studies that aim at disentangling the role of various floral traits during the various stages of pollination and the potential for independent adaption might shed light on the multi-selection pressures on floral traits.

## Conclusion

The results presented here based on the pollen-transfer networks of nine spatially and temporally separated meadow communities in Southern Germany show that our set of floral traits are not well correlated with the quantity of pollen transfer but rather with its quality. We have detected a high impact of floral abundance on network properties, a finding that highlights the importance of community-specific properties and suggests geographic differences. In general, our results indicate that floral traits, known to mediate flower-visitor interactions, can elucidate who is sharing pollen with whom but does not give information about the intensity and quantity of HP transfer, which more likely depends on other floral traits and the identity of flower visitors.

## Material and methods

### Study sites

Our study was conducted between May and July 2018 in three protected dry nutrient-poor limestone grasslands (juniper heathlands), namely Eselsburger Tal (48°36′13.2408"N 10°10′39.6768"O), Hirschtal (48°41′44.1204"N 10°2′8.6388"O) and Reichenbach (Haarberg/Wasserberg; 48°37′27.2316"N 9°43′59.3796"O), located on the Swabian Alb, Germany. In each grassland, we used three plots (50 × 50 m) established by the BienABest project (https://www.bienabest.de/) and conducted three sampling rounds (early summer, mid-summer and late summer): Eselsburger Tal 11-May, 12-July and 30-July; Hirschtal 22-May, 2-July and 25-July; Reichenbach 18-May, 9-July and 24-July-2018. Sampling was conducted on sunny days and restricted to weekdays based on permit regulations. Thus, in total, we sampled nine communities at three locations and time points.

### Flower abundance and stigma collection

In the centre of each plot, we established one transect (2 m × 50 m), identified all flowering plant species therein and counted the number of flowers per species. For plant species with a large number of small flowers, e.g. Apiaceae, we counted all inflorescences and multiplied them with the mean number of flowers from five inflorescences. To measure stigmatic pollen loads accumulated across the full floral life time, we collected five stigmas of senescent flowers of five different individuals per flowering plant species on each plot (median number of stigmas per species and location = 10). Plant individuals were distributed across the full transect length in a maximum distance of 10 m, when possible. Because of restrictions for protected plant species and for plant species for which no senescent flowers were found, our sample completeness of plant species in each location ranged from 78 to 100% (mean ± SD: 86.1 ± 7.3%; Table 1; Additional file 1-6). Additionally, we collected flowers in anthesis for each species and established a reference pollen collection. Collected stigmas were stored in Eppendorf tubes in a freezer at -20 °C until further preparation.

### Pollen evaluation

For the enumeration and determination of stigmatic HP and CP load, we followed commonly used non-genetic methods [26, 27, 30, 37, 38]. We placed each stigma in a droplet of Alexanders’ stain [39] on a microscopic slide, covered each preparation with a coverslip and sealed the preparation with transparent nail varnish under a stereoscopic microscope (Stemi 2000-CS; Carl ZEISS AG). Subsequently, the microscopic slides where incubated 48 h at 50 °C. Pollen for the reference collection was prepared accordingly. For protected species, our reference collection was supplemented by images found in the open source database PalDat (PalDat, 2000). For the reference collection, we took photographs from pollen grains at various planes and measured pollen size for all species under the light microscope (Scope.A1; Carl ZEISS AG) at 63–100 × magnification.

Pollen was evaluated under the same light microscope. All CP and HP were enumerated and determined by using the prepared reference pollen collection. In cases in which the identification of HP origin was not possible from our reference collection, we categorized the pollen as “unknown” (unidentified pollen was found on 9% of all stigmas and accounted for 10% of all sampled HP grains). If the identification of pollen was unclear between two species, we assumed the species with the higher abundance as being the origin.

### Floral trait measurements

For each plant species (total *n* = 52), we collected five individuals per species from one or two locations. For five species, we only collected three or four individuals. Plant species under protection were not collected (Additional file 1-6). We measured seven morphological floral traits known to mediate plant-pollinator interaction [15, 21, 23, 25] by using a caliper rule under a stereomicroscope (Stemi 2000-CS; Carl ZEISS AG): (1) stamen length [mm], (2) inflorescence diameter [mm], (3) nectar tube width [mm], (4) nectar tube depth [mm] (both zero if no nectar tube was present), (5) display size of flower [mm] (maximum distance between apical ends of two petals) and (6) style length [mm].

### Bee species richness and abundance

Data on bee species and abundance were provided by the BienABest project (www.bienabest.de). Here, at each location (*n* = 3), each plot (*n* = 3) and each sampling date (*n* = 3), wild bees were sampled during one day once before and once after noon by means of walking along the available resources for 25 min [40]. Each bee observed was noted and the bees that could not be identified to species level at first sight were caught and identified in the laboratory. This sampling procedure allowed us to determine differences in bee abundance between sites but may not have represented accurate absolute numbers of bees per site. For example, one day per week (or even fewer) is often used in monitoring programmes when determining abundance differences between sites or years [41–43]. Further, if the flower longevity in our grasslands is 1 to 1 1/2 weeks, an estimation of the abundance at one particular time should provide an adequate approximation of abundance within this timeframe.

### Statistical analysis

For analysing the pollen transfer based on stigmatic HP loads within the different communities (all three plots were summarized), we created unipartite directed networks by using the open-source software *Gephi* version 0.9.2 [44]. Each established link between two plants was defined as pollen grains from one plant species (a) found on the stigma of another plant species (b). Each link was weighted by the number of pollen grains received by the receptor species (b) from the donor species (a). In each network, we calculated the in- and out-degree number for each species. The in-degree was defined as the number of incoming links, whereas the out-degree was defined as the number of outgoing links. Based on the in- and out-degree values, we identified whether the plant species act as a hub-donor (in-degree < out-degree), a hub-receptor (in-degree > out-degree) or a neutral species (in-degree = out-degree) [45]. Further, the weighted in- and out-degree values were calculated [44], which weighted each link by its intensity, i.e. pollen grain number. Lastly, the modularity was calculated using the optimization algorithm of Blondel et al. [46]. We used a resolution factor of 0.7 [47] and weighted links according the amount of HP. As modularity and species composition in each module can vary between runs, the algorithm was run 25 times for each network. For EBI, HTI and HTII, all runs resulted in the same number of modules, modularity and species composition in each module. For EBII and EBIII, 20 runs resulted in the same results and, for RBI and RBII, 22 and 23 runs. For all these networks, we considered the results that appeared > 80% as the most likely ones. For RBIII, two solutions were equally likely, both appearing 11 times. Both had a similar modularity of 0.474 and 0.472. However, they differed in the module assignment of three species (Table 2). Analyses were calculated with both. All parameters of plant-plant networks and graphs were carried out in *Gephi* version 0.9.2 [44].

Further statistical analyses were performed in *R* version 3.4.4 [48]. To test the relationship between in- and out-degree values and measured floral traits and flower abundance (question I), we performed generalized linear mixed models (GLMMs) with a beta distribution. The mean stamen length, inflorescence diameter, nectar tube depth, nectar tube width, display size, style length or flower abundance were included as the fixed factor and the community and plant species as the random factor. Flower abundance was ln-transformed to reduce the importance of highly abundant small flowers, e.g. Apiaceae. For standardization, in- and out-degree values were divided by the number of flowering plant species per community and transformed according to Cribari-Neto & Zeileis [49] for beta regression. All models were calculated using the *glmmTMB*-package [50] with the *glmmTMB*-function. To test the relationship between weighted in- and out-degree values and measured floral traits and flower abundance (question I), we performed Poisson-distributed GLMMs by using the *lme4*-package [51] with the *glmer*-function. The same model structure as describe for in- and out-degree values was used. The fit for each model was validated using the *DHARMa*-package [52] as described in the package vignette.

To test whether the modularity of plant-plant networks was affected by community parameters, i.e. bee species richness, abundance, diversity, plant species richness, flower abundance (ln-transformed) and diversity (question II), we used linear regression models (LM). Bee and flower diversities were calculated as Shannon–Wiener indices by using the *diversity*-function of the *vegan*-package [53]. If necessary, modularity was ln + 1-transformed to achieve normality.

To address whether floral traits could explain the module classification of the plant species (question III), we used random forest analysis by using the *randomForest*-package [54]. Random forest is a machine learning algorithm [55]; here, it assigned plant individuals to the predefined modules in multiple iterations based on the measured floral traits and estimated the importance of each floral trait for the correct classification of each plant individual. For this analysis, we used ntree = 10,000 bootstrap replicates drawn with the mtry = 2 variable randomly selected at each node. To identify the most important variable for classification, we used the *importance*-function of the same package.

To test whether floral traits and floral abundance are linked to the ratio of the received HP percentage (question IV), we performed binomial GLMMs, as the beta distribution showed a poor model fit. All models were calculated using the *lme4*-package [51] with the *glmer*-function. The significance for each fixed factor was determined via the likelihood ratio test by comparing the model including the fixed and random factors with the model only including random factors. If the model convergence failed when we used the default arguments, we first reran the model by restarting from the previous fit, this increased the maximum number of iterations. In those cases in which the restart did not result in model convergence, we replaced the default optimizer in the second phase with the bobyqa-optimizer. This step always resulted in model convergence. The relative contribution of the model factors to the variation in the interaction patterns was estimated by calculating *R*^*2*^_*marginal*_ and *R*^*2*^_*conditional*_ [56]. Each model was validated as described above.

To estimate sample completeness, we calculated sample and incidence-based rarefaction curves for each community by using the iNEXT-package [57]. Overall, for most species, curves did not reach an asymptote and curves for the same species differed between communities. However, within-community extrapolated curves showed no changes in the relative positions of the various species compared with interpolated curves. Further, none of the overlapping curves indicated that the relative position of the species to each other would have been stable with further sampling. As the sampling was the same in all communities, the absolute values might be biased, whereas the relative values between communities should not be affected, e.g. the absolute values of modularity compared with correlations of modularity having community properties. Therefore, the results can be interpreted accordingly within our study. Full results are shown in Additional file 1-7.

## Supplementary information

**Additional file 1.** **1.** Correlation between floral traits and in- and out-degree. **2.** Correlation between floral traits and weighted in- and out-degree. **3.** Correlations between modularity and community parameters. **4.** Results of random forest analysis for modularity and floral traits. **5.** Correlations between proportion of HP and floral traits. **6.** Flower abundance and plant species sampled. **7.** Rarefaction curves for each community.

## Data Availability

The data used in this paper are available on Figshare 10.6084/m9.figshare.13013282.
