# Supplementary material for: Floral traits are associated with the quality but not quantity of heterospecific stigmatic pollen loads
Source: BMC Ecol. 2020 Oct 6;20:54. doi: 10.1186/s12898-020-00323-5 (PMC7539470; doi:10.1186/s12898-020-00323-5)
Supplement: Supplementary file 1 — Additional file 1. 1. Correlation between floral traits and in- and out-degree. 2. Correlation between floral traits and weighted in- and out-degree. 3. Correlations between modularity and community parameters. 4. Results of random forest analysis for modularity and floral traits. 5. Correlations between proportion of HP and floral traits. 6. Flower abundance and plant species sampled. 7. Rarefaction curves for each community. [file 12898_2020_323_MOESM1_ESM.docx]

**Additional file 1: Floral traits are associated with the quantity but not quality of heterospecific stigmatic pollen loads**

Manon A. Peuker^1^, Hannah Burger^1^, Sabrina Krausch^1^, Ulrich Neumüller^1^, Manfred Ayasse^1^, Jonas Kuppler^1,^*

^1^Institute of Evolutionary Ecology and Conservation Genomics, Ulm University, Ulm, Germany

*corresponding author: Jonas Kuppler, Institute of Evolutionary Ecology and Conservation Genomics, Ulm University, Albert-Einstein-Allee 11, 89081 Ulm, Germany; phone: 0049-731-5022665; fax: 0049-731-5022683; e-mail: jonas.kuppler@mail.de

BMC Ecology (2020)

*Additional file 1-1: Correlation between floral traits and in- and out-degree*

**Additional file 1-1**. Generalized linear mixed models (GLMM) for in- and out-degree and measured floral traits, i.e. stamen length, inflorescence diameter, nectar tube depth, nectar tube width, display size, stigma length, and flower abundance (ln-transformed). In- and out-degree are expressed as percentage of total number of plant species in each community and transformed following (Cribari-Neto & Zeileis 2010). Beta-distributed GLMMs included one floral traits or floral abundance as fixed factor and plant species and community as random factor; models including stamen length as fixed factor also included squared stamen length. Model coefficients (log-link) and *p*-values are given.

|  | **Term** | **Coefficient** | **Standard Error** | ***z*** |
| --- | --- | --- | --- | --- |
| **In-degree** |  |  |  |  |
|  |  |  |  |  |
|  |  |  |  |  |
|  | *Intercept* | -1.37 | 0.232 | -5.89*** |
|  | *Stamen length^+^* | -0.041 | 0.151 | -0.271 |
|  | *Stamen length^2^+^* | -0.31 | 0.185 | -1.676 |
|  |  |  |  |  |
|  | *Intercept* | -1.66 | 0.177 | -9.394*** |
|  | *Inflorescence diameter^+^* | -0.008 | 0.141 | -0.055 |
|  |  |  |  |  |
|  | *Intercept* | -1.658 | 0.177 | -9.379*** |
|  | *Nectar tube depth^+^* | -0.117 | 0.147 | -0.792 |
|  |  |  |  |  |
|  | *Intercept* | -1.667 | 0.173 | -9.66*** |
|  | *Nectar tube width^+^* | 0.164 | 0.122 | 1.344 |
|  |  |  |  |  |
|  | *Intercept* | -1.671 | 0.182 | -9.192*** |
|  | *Display size^+^* | 0.18 | 0.133 | 1.357 |
|  |  |  |  |  |
|  | *Intercept* | -1.665 | 0.174 | -9.542*** |
|  | *Style length^+^* | -0.072 | 0.151 | -0.478 |
|  |  |  |  |  |
|  | *Intercept* | -1.668 | 0.313 | -5.335** |
|  | *Flower abundance* | 0.002 | 0.05 | 0.049 |
|  |  |  |  |  |
| **Out-degree** |  |  |  |  |
|  |  |  |  |  |
|  | *Intercept* | -1.965 | 0.145 | -13.54*** |
|  | *Stamen length* | -0.274 | 0.135 | -2.031* |
|  |  |  |  |  |
|  | *Intercept* | -1.95 | 0.148 | -13.171*** |
|  | *Inflorescence diameter* | -0.014 | 0.128 | -0.112 |
|  |  |  |  |  |
|  | *Intercept* | -1.95 | 0.144 | -13.481*** |
|  | *Nectar tube depth* | -0.221 | 0.132 | -1.671 |
|  |  |  |  |  |
|  | *Intercept* | -1.949 | 0.147 | -13.281*** |
|  | *Nectar tube width* | -0.133 | 0.121 | -1.098 |
|  |  |  |  |  |
|  | *Intercept* | -1.943 | 0.147 | -13.197*** |
|  | *Display size* | -0.082 | 0.128 | -0.643 |
|  |  |  |  |  |
|  | *Intercept* | -1.968 | 0.146 | -13.477*** |
|  | *Style length^+^* | -0.257 | 0.136 | -1.892 |
|  |  |  |  |  |
|  | *Intercept* | -2.60 | 0.298 | -8.737*** |
|  | *Flower abundance* | 0.121 | 0.049 | 2.481* |

^+^variable were centered by subtracting the column means of x from their corresponding columns and scaled by dividing the (centered) columns of x by their standard deviations

* *p* < 0.05, ** *p* < 0.01, *p* < 0.001***; significant values for likelihood ratio tests are highlighted in bold.

*Additional file 1-2: Correlation between floral traits and weighted in- and out-degree*

**Additional file 1-2**. Generalized linear mixed models (GLMM) for weighted in- and out-degree and measured floral traits, i.e. stamen length, inflorescence diameter, nectar tube depth, nectar tube width, display size, stigma length, and flower abundance (ln-transformed). Weighted In- and out-degree were calculated for pollen transfer plant-plant networks in *Gephi* version 0.9.2 (Bastian *et al.* 2009). Poisson-distributed GLMMs included one floral traits or floral abundance as fixed factor and plant species and community as random factor; models including stamen and style length as fixed factor also included squared stamen and style length to meet model assumptions. Model coefficients (log-link) and *p*-values are given.

|  | **Term** | **Coefficient** | **Standard Error** | ***z*** |
| --- | --- | --- | --- | --- |
| **In-degree** |  |  |  |  |
|  |  |  |  |  |
|  |  |  |  |  |
|  | *Intercept* | 2.208 | 0.924 | 2.389* |
|  | *Stamen length* | 0.125 | 0.308 | 0.405 |
|  | *Stamen length^2* | -0.006 | 0.021 | -0.262 |
|  |  |  |  |  |
|  | *Intercept* | 2.566 | 0.679 | 3.780*** |
|  | *Inflorescence diameter* | 0.004 | 0.020 | 0.214 |
|  |  |  |  |  |
|  | *Intercept* | 3.077 | 0.496 | 6.200*** |
|  | *Nectar tube depth* | -0.093 | 0.074 | -1.259 |
|  |  |  |  |  |
|  | *Intercept* | 2.360 | 0.417 | 5.656*** |
|  | *Nectar tube width* | 0.167 | 0.083 | 2.004* |
|  |  |  |  |  |
|  | *Intercept* | 2.071 | 0.503 | 4.118*** |
|  | *Display size* | 0.045 | 0.238 | 1.907 |
|  |  |  |  |  |
|  | *Intercept* | 2.421 | 0.639 | 3.787*** |
|  | *Style length* | 0.051 | 0.124 | 0.409 |
|  | *Style length^2* | -0.001 | 0.005 | -0.233 |
|  |  |  |  |  |
|  | *Intercept* | 2.485 | 0.397 | 6.256*** |
|  | *Flower abundance* | 0.039 | 0.017 | 2.344* |
|  |  |  |  |  |
| **Out-degree** |  |  |  |  |
|  |  |  |  |  |
|  | *Intercept* | 2.943 | 0.714 | 4.120*** |
|  | *Stamen length* | -0.162 | 0.087 | -1.859 |
|  |  |  |  |  |
|  | *Intercept* | 2.203 | 0.872 | 2.326* |
|  | *Inflorescence diameter* | -0.003 | 0.026 | -0.152 |
|  |  |  |  |  |
|  | *Intercept* | 2.457 | 0.614 | 4.004*** |
|  | *Nectar tube depth* | -0.125 | 0.093 | -1.343 |
|  |  |  |  |  |
|  | *Intercept* | 2.342 | 0.531 | 4.414*** |
|  | *Nectar tube width* | -0.230 | 0.137 | -1.678 |
|  |  |  |  |  |
|  | *Intercept* | 2.388 | 0.642 | 3.722*** |
|  | *Display size* | -0.034 | 0.032 | -1.074 |
|  |  |  |  |  |
|  | *Intercept* | 2.530 | 0.630 | 4.016*** |
|  | *Style length* | -0.082 | 0.057 | -1.429 |
|  |  |  |  |  |
|  | *Intercept* | 1.429 | 0.463 | 3.086** |
|  | *Flower abundance* | 0.101 | 0.018 | 5.535*** |

^+^variable were centered by subtracting the column means of x from their corresponding columns and scaled by dividing the (centered) columns of x by their standard deviations

* *p* < 0.05, ** *p* < 0.01, *p* < 0.001***; significant values for likelihood ratio tests are highlighted in bold.

*Additional file 1-3: Correlations between modularity and community parameters*

**Additional file 1-3**. Linear models for modularity of pollen-transfer plant-plant networks and community parameters. Community parameters are: bee species richness, bee abundance, bee diversity, plant species richness, flower abundance (ln-transformed) and flower diversity. Diversity was measured as Shannon-Wiener index. Model coefficients, *F*-statistics and adjusted *R^2^* are given.

| **Term** | **Coefficient** | **Standard Error** | ***t*** | ***F_1,7_*** | **adjusted *R^2^*** |
| --- | --- | --- | --- | --- | --- |
|  |  |  |  |  |  |
| *Intercept* | 0.029 | 0.17 | 0.168 |  |  |
| *Bee species richness* | 0.01 | 0.005 | 1.909 | 3.646 | 0.25 |
|  |  |  |  |  |  |
| *Intercept* | 0.15 | 0.068 | 2.221 |  |  |
| *Bee abundance* | 0.001 | 0.0005 | 2.386* | **5.691*** | 0.37 |
|  |  |  |  |  |  |
| *Intercept* | -0.522 | 0.70 | -0.74 |  |  |
| *Bee diversity*^+^ | 0.344 | 0.281 | 1.226 | 1.503 | 0.06 |
|  |  |  |  |  |  |
|  |  |  |  |  |  |
| *Intercept* | -0.002 | 0.061 | -0.03 |  |  |
| *Plant species richness*^+^ | 0.019 | 0.004 | 5.085* | **25.86**** | 0.76 |
|  |  |  |  |  |  |
| *Intercept* | -0.83 | 0.308 | -2.706* |  |  |
| *Flower abundance* | 0.122 | 0.032 | 3.83** | **14.67**** | 0.63 |
|  |  |  |  |  |  |
| *Intercept* | -0.412 | 0.096 | -4.277** |  |  |
| *Flower diversity* | 0.307 | 0.039 | 7.963*** | **63.41***** | 0.89 |

^+^modularity was ln+1 transformed to achieve normality

* *p* < 0.05, ** *p* < 0.01, *p* < 0.001***; significant values for likelihood ratio tests are highlighted in bold.

*Additional file 1-4: Results of random forest analysis for modularity and floral traits*

**Additional file 1-4.1**. Classification of plant species into modules based on floral traits for eight communities. Shown are the number of correctly assigned plant species (highlighted in bold), the proportional class error for each module and the total OOB estimate of the error rate. Numbers in community names show sampling date: I = early summer, II = mid summer, III = late summer. Eselsburger Tal I is not shown as no modules were detected. Modularity was calculated for pollen transfer plant-plant networks in *Gephi* version 0.9.2 (Bastian *et al.* 2009).

| **Community** |  |  |  |  |  |  | **Class** **error** |
| --- | --- | --- | --- | --- | --- | --- | --- |
| *Eselsburger Tal II* | Module | Module | Module | Module | Module | Module |  |
|  | A | B | C | D | E | F |  |
| Module A | **21** | 1 | 1 | 1 | 0 | 0 | 0.09 |
| Module B | 0 | **15** | 0 | 0 | 0 | 0 | 0.00 |
| Module C | 1 | 0 | **39** | 0 | 0 | 0 | 0.03 |
| Module D | 0 | 1 | 0 | **23** | 0 | 0 | 0.04 |
| Module E | 1 | 0 | 0 | 0 | **4** | 0 | 0.20 |
| Module F | 0 | 0 | 0 | 0 | 0 | **10** | 0.00 |
| OOB estimate of error rate: 4.27% | | | | | | | |
|  |  |  |  |  |  |  |  |
| *Eselsburger Tal III* | Module | Module |  |  |  |  |  |
|  | A | B |  |  |  |  |  |
| Module A | **30** | 0 |  |  |  |  | 0.00 |
| Module B | 0 | **14** |  |  |  |  | 0.00 |
| OOB estimate of error rate: 0.00% | | | | | | | |
|  |  |  |  |  |  |  |  |
| *Hirschtal I* | Module | Module |  |  |  |  |  |
|  | A | B |  |  |  |  |  |
| Module A | **14** | 1 |  |  |  |  | 0.07 |
| Module B | 0 | **20** |  |  |  |  | 0.00 |
| OOB estimate of error rate: 2.86% | | | | | | | |
|  |  |  |  |  |  |  |  |
| *Hirschtal II* | Module | Module | Module | Module |  |  |  |
|  | A | B | C | D |  |  |  |
| Module A | **18** | 1 | 1 | 0 |  |  | 0.10 |
| Module B | 1 | **23** | 1 | 5 |  |  | 0.23 |
| Module C | 0 | 1 | **17** | 0 |  |  | 0.06 |
| Module D | 0 | 5 | 0 | **10** |  |  | 0.33 |
| OOB estimate of error rate: 18.07% | | | | | | | |
|  |  |  |  |  |  |  |  |
| *Hirschtal III* | Module | Module | Module | Module |  |  |  |
|  | A | B | C | D |  |  |  |
| Module A | **38** | 1 | 1 | 0 |  |  | 0.05 |
| Module B | 0 | **22** | 0 | 0 |  |  | 0.00 |
| Module C | 3 | 0 | **12** | 0 |  |  | 0.20 |
| Module D | 0 | 1 | 0 | **9** |  |  | 0.10 |
| OOB estimate of error rate: 6.90% | | | | | | | |
|  |  |  |  |  |  |  |  |
| *Reichenbach I* | Module | Module | Module |  |  |  |  |
|  | A | B | C |  |  |  |  |
| Module A | **12** | 1 | 0 |  |  |  | 0.08 |
| Module B | 1 | **19** | 0 |  |  |  | 0.05 |
| Module C | 1 | 1 | **8** |  |  |  | 0.20 |
| OOB estimate of error rate: 9.30% | | | | | | | |
|  |  |  |  |  |  |  |  |
| *Reichenbach II* | Module | Module | Module | Module | Module |  |  |
|  | A | B | C | D | E |  |  |
| Module A | **6** | 0 | 1 | 3 | 0 |  | 0.40 |
| Module B | 1 | **35** | 1 | 0 | 0 |  | 0.05 |
| Module C | 1 | 1 | **25** | 2 | 1 |  | 0.17 |
| Module D | 0 | 1 | 1 | **18** | 0 |  | 0.10 |
| Module E | 0 | 1 | 0 | 0 | **9** |  | 0.10 |
| OOB estimate of error rate: 13.08% | | | | | | | |
|  |  |  |  |  |  |  |  |
| *Reichenbach III* | Module | Module | Module | Module |  |  |  |
|  | A | B | C | D |  |  |  |
| Module A | **32** | 3 | 0 | 0 |  |  | 0.09 |
| Module B | 2 | **52** | 0 | 1 |  |  | 0.05 |
| Module C | 0 | 0 | **9** | 0 |  |  | 0.00 |
| Module D | 1 | 1 | 0 | **26** |  |  | 0.07 |
| OOB estimate of error rate: 6.30% | | | | | | | |
| *Reichenbach III 2* | Module | Module | Module | Module | Module |  |  |
|  | A | B | C | D | E |  |  |
| Module A | **32** | 3 | 0 | 0 | 0 |  | 0.09 |
| Module B | 2 | **37** | 0 | 1 | 0 |  | 0.08 |
| Module C | 0 | 1 | **8** | 0 | 0 |  | 0.11 |
| Module D | 1 | 1 | 0 | **26** | 0 |  | 0.07 |
| Module E | 0 | 2 | 0 | **0** | **13** |  | 0.13 |
| OOB estimate of error rate: 8.66% | | | | | | | |

**Additional file 1-4.2** Module assignment for each plant species in each of the nine communities. Sampling date (=Time): early summer (I), mid summer (II), late summer (III). For RB III differences in module affiliation between two equally likely solutions of the modularity algorithm (Blondel *et al.* 2008)are shown. Solutions one with four modules / solution two with five modules as one module was split into two.

| **Location** | **Time** | **Plant species** | **Module assignment** |
| --- | --- | --- | --- |
| Eselsburger Tal | I | *Cerastium arvense* | A |
| Eselsburger Tal | I | *Euphorbia cyparissias* | A |
| Eselsburger Tal | I | *Lotus corniculatus* | A |
| Eselsburger Tal | I | *Polygala vulgaris* | A |
| Eselsburger Tal | I | *Tripleurospermum inodorum* | A |
| Eselsburger Tal | II | *Heliantemum nummularium* | A |
| Eselsburger Tal | II | *Lotus corniculatus* | A |
| Eselsburger Tal | II | *Leucanthemum vulgare* | A |
| Eselsburger Tal | II | *Trifolium dubium* | A |
| Eselsburger Tal | II | *Trifolium pratense* | A |
| Eselsburger Tal | II | *Ononis spinosa* | B |
| Eselsburger Tal | II | *Scabiosa columbaria* | B |
| Eselsburger Tal | II | *Sedum acre* | B |
| Eselsburger Tal | II | *Agrimonia eupatoria* | C |
| Eselsburger Tal | II | *Asperula cynanchica* | C |
| Eselsburger Tal | II | Asteraceae sp2 | C |
| Eselsburger Tal | II | *Thymus pulegioides* | C |
| Eselsburger Tal | II | *Anthemis tinctoria* | C |
| Eselsburger Tal | II | *Campanula rotundifolia* | C |
| Eselsburger Tal | II | *Dianthus carthusianorum* | C |
| Eselsburger Tal | II | *Galium verum* | C |
| Eselsburger Tal | II | *Trifolium campestre* | C |
| Eselsburger Tal | II | *Centaurea jacea* | D |
| Eselsburger Tal | II | *Daucus carota* | D |
| Eselsburger Tal | II | *Prunella vulgaris* | D |
| Eselsburger Tal | II | Papaver sp | D |
| Eselsburger Tal | II | *Cirsium arvense* | D |
| Eselsburger Tal | II | *Melampyrum arvense* | D |
| Eselsburger Tal | II | *Teucrium montanum/chamaedrys* | E |
| Eselsburger Tal | II | *Echium vulgare* | E |
| Eselsburger Tal | II | *Coronilla varia* | F |
| Eselsburger Tal | II | *Teucrium chamaedrys* | F |
| Eselsburger Tal | III | *Campanula rotundifolia* | A |
| Eselsburger Tal | III | *Aethusa cynapium* | A |
| Eselsburger Tal | III | *Scabiosa columbaria* | A |
| Eselsburger Tal | III | *Galium verum* | A |
| Eselsburger Tal | III | *Silene vulgaris* | A |
| Eselsburger Tal | III | *Thymus pulegioides* | A |
| Eselsburger Tal | III | *Cirsium arvense* | B |
| Eselsburger Tal | III | *Centaurea jacea* | B |
| Eselsburger Tal | III | *Ononis spinosa* | B |
| Hirschtal | I | *Salvia pratensis* | A |
| Hirschtal | I | *Veronica chamaedrys* | A |
| Hirschtal | I | *Ajuga reptans* | A |
| Hirschtal | I | *Euphorbia cyparissias* | B |
| Hirschtal | I | *Tripleurospermum inodorum* | B |
| Hirschtal | I | Ranunculus sp | B |
| Hirschtal | I | *Lotus corniculatus* | B |
| Hirschtal | II | *Agrimonia eupatoria* | A |
| Hirschtal | II | *Anthemis tinctoria* | A |
| Hirschtal | II | *Dianthus carthusianorum* | A |
| Hirschtal | II | *Origanum vulgare* | A |
| Hirschtal | II | *Scabiosa columbaria* | A |
| Hirschtal | II | *Achillea millefolium* | B |
| Hirschtal | II | *Galium verum* | B |
| Hirschtal | II | *Thymus pulegioides* | B |
| Hirschtal | II | *Asperula cynanchica* | B |
| Hirschtal | II | Asteraceae sp | B |
| Hirschtal | II | *Hypericum perforatum* | B |
| Hirschtal | II | *Tripleurospermum inodorum* | B |
| Hirschtal | II | *Teucrium montanum/chamaedrys* | B |
| Hirschtal | II | *Lotus corniculatus* | C |
| Hirschtal | II | *Cichorium intybus* | C |
| Hirschtal | II | *Coronilla varia* | C |
| Hirschtal | II | *Heliantemum nummularium* | C |
| Hirschtal | II | *Stachys recta* | C |
| Hirschtal | II | *Salvia verticillata* | D |
| Hirschtal | II | *Trifolium pratense* | D |
| Hirschtal | II | *Teucrium chamaedrys* | D |
| Hirschtal | III | *Aethusa cynapium* | A |
| Hirschtal | III | *Achillea millefolium* | A |
| Hirschtal | III | *Scabiosa columbaria* | A |
| Hirschtal | III | *Thymus pulegioides* | A |
| Hirschtal | III | *Galium verum* | A |
| Hirschtal | III | *Dianthus carthusianorum* | A |
| Hirschtal | III | *Hypericum perforatum* | A |
| Hirschtal | III | *Prunella vulgaris* | A |
| Hirschtal | III | *Coronilla varia* | A |
| Hirschtal | III | *Agrimonia eupatoria* | B |
| Hirschtal | III | *Lotus corniculatus* | B |
| Hirschtal | III | *Cirsium arvense* | B |
| Hirschtal | III | *Heliantemum nummularium* | B |
| Hirschtal | III | *Trifolium pratense* | B |
| Hirschtal | III | *Asperula cynanchica* | C |
| Hirschtal | III | *Origanum vulgare* | C |
| Hirschtal | III | *Daucus carota* | C |
| Hirschtal | III | *Clinopodium vulgare* | D |
| Hirschtal | III | *Trifolium dubium* | D |
| Reichenbach | I | *Ajuga reptans* | A |
| Reichenbach | I | *Euphorbia cyparissias* | A |
| Reichenbach | I | *Vincetoxicum hirundinaria* | A |
| Reichenbach | I | *Lotus corniculatus* | B |
| Reichenbach | I | *Trifolium repens* | B |
| Reichenbach | I | *Tripleurospermum inodorum* | B |
| Reichenbach | I | *Polygala vulgaris* | B |
| Reichenbach | I | *Onobrychis viciifolia* | C |
| Reichenbach | I | *Salvia pratensis* | C |
| Reichenbach | II | Asteraceae sp2 | A |
| Reichenbach | II | *Centaurea jacea* | A |
| Reichenbach | II | *Asperula cynanchica* | B |
| Reichenbach | II | *Agrimonia eupatoria* | B |
| Reichenbach | II | *Campanula rotundifolia* | B |
| Reichenbach | II | *Cichorium intybus* | B |
| Reichenbach | II | *Cirsium arvense* | B |
| Reichenbach | II | *Daucus carota* | B |
| Reichenbach | II | *Heliantemum nummularium* | B |
| Reichenbach | II | *Aster amellus* | B |
| Reichenbach | II | *Senecio erucifolius* | B |
| Reichenbach | II | *Echium vulgare* | B |
| Reichenbach | II | *Leucanthemum vulgare* | C |
| Reichenbach | II | *Aethusa cynapium* | C |
| Reichenbach | II | *Galium verum* | C |
| Reichenbach | II | *Origanum vulgare* | C |
| Reichenbach | II | *Silene vulgaris* | C |
| Reichenbach | II | *Medicago falcata* | C |
| Reichenbach | II | *Lotus corniculatus* | D |
| Reichenbach | II | *Cichorium intibus* | D |
| Reichenbach | II | *Ononis spinosa* | D |
| Reichenbach | II | *Trifolium pratense* | D |
| Reichenbach | II | *Prunella vulgaris* | E |
| Reichenbach | II | *Melampyrum arvense* | E |
| Reichenbach | III | *Agrimonia eupatoria* | A |
| Reichenbach | III | *Campanula rotundifolia* | A |
| Reichenbach | III | *Daucus carota* | A |
| Reichenbach | III | *Sedum acre* | A |
| Reichenbach | III | *Cichorium intibus* | A |
| Reichenbach | III | *Leucanthemum vulgare* | A |
| Reichenbach | III | *Origanum vulgare* | A |
| Reichenbach | III | *Senecio erucifolius* | A |
| Reichenbach | III | *Asperula cynanchica* | B / E |
| Reichenbach | III | Asteraceae sp2 | B |
| Reichenbach | III | *Anthemis tinctoria* | B / E |
| Reichenbach | III | *Scabiosa columbaria* | B |
| Reichenbach | III | *Thymus pulegioides* | B |
| Reichenbach | III | *Tripleurospermum inodorum* | B |
| Reichenbach | III | *Centaurea jacea* | B |
| Reichenbach | III | *Galium verum* | B |
| Reichenbach | III | *Ononis spinosa* | B |
| Reichenbach | III | *Prunella vulgaris* | B / E |
| Reichenbach | III | *Cichorium intybus* | B |
| Reichenbach | III | *Teucrium chamaedrys* | B |
| Reichenbach | III | *Cirsium arvense* | C |
| Reichenbach | III | *Aster amellus* | C |
| Reichenbach | III | *Lotus corniculatus* | D |
| Reichenbach | III | *Coronilla varia* | D |
| Reichenbach | III | *Trifolium pratense* | D |
| Reichenbach | III | *Trifolium dubium* | D |
| Reichenbach | III | *Heliantemum nummularium* | D |
| Reichenbach | III | *Dianthus carthusianorum* | D |
| Reichenbach | III | *Medicago falcata* | D |

*Additional file 1-5: Correlations between proportion of HP and floral traits*

**Additional file 1-5**. Generalized linear mixed models (GLMM) for proportion of HP (percentage) and measured floral traits, i.e. stamen length, inflorescence diameter, nectar tube depth, nectar tube width, display size, style length, and flower abundance (ln-transformed). Binominal-distributed GLMMs included one floral traits or floral abundance as fixed factor and plant species and community as random factor. Model coefficients and results of likelihood ratio tests (comparing model including fixed and random factors and model including only random factors) are given.

| **Term** | **Coefficient** | **Standard Error** | ***z*** | **Likelihood ratio test** $X_{1}^{2}$ | ***R^2^_marginal_*** | ***R^2^_conditional_*** |
| --- | --- | --- | --- | --- | --- | --- |
| *Intercept* | -3.21 | 0.71 | -4.522*** |  |  |  |
| *Stamen length* | 0.002 | 0.092 | 0.021 | 0.0002 | 6e-6 | 0.64 |
|  |  |  |  |  |  |  |
| *Intercept* | -4.061 | 0.822 | -4.938*** |  |  |  |
| *Inflorescence diameter* | 0.031 | 0.026 | 1.218 | 1.44 | 0.017 | 0.64 |
|  |  |  |  |  |  |  |
| *Intercept* | -2.740 | 0.581 | -4.712*** |  |  |  |
| *Nectar tube depth* | -0.109 | 0.099 | -1.094 | 1.185 | 0.015 | 0.65 |
|  |  |  |  |  |  |  |
| *Intercept* | -3.177 | 0.474 | -6.706*** |  |  |  |
| *Nectar tube width* | -0.009 | 0.120 | -0.075 | 0.005 | 6e-5 | 0.64 |
|  |  |  |  |  |  |  |
| *Intercept* | -3.473 | 0.611 | -5.682*** |  |  |  |
| *Display size* | 0.021 | 0.034 | 0.613 | 0.369 | 0.004 | 0.64 |
|  |  |  |  |  |  |  |
| *Intercept* | -3.334 | 0.669 | -5.517*** |  |  |  |
| *Style length* | 0.019 | 0.061 | 0.314 | 0.096 | 0.001 | 0.64 |
|  |  |  |  |  |  |  |
| *Intercept* | -2.46 | 0.432 | -5.690*** |  |  |  |
| *Floral abundance* | -0.142 | 0.019 | -7.570*** | **57.99***** | 0.01 | 0.65 |

* *p* < 0.05, ** *p* < 0.01, *p* < 0.001***; significant values for likelihood ratio tests are highlighted in bold.

*Additional file 1-6: Flower abundance and plant species sampled*

**Additional file 1-6**. Flower abundance and plant species sampled separated by location and sampling date. Sampling date (=Time): early summer (I), mid summer (II), late summer (III). + indicate that plant species was sampled, - indicate that no stigmas were collected.

| **Location** | **Time** | **Plant species** | **Flower abundance** | **Number of samples** |
| --- | --- | --- | --- | --- |
| Eselsburger Tal | I | *Cerastium arvense* | 109 | + |
| Eselsburger Tal | I | *Euphorbia cyparissias* | 1468 | + |
| Eselsburger Tal | I | *Lotus corniculatus* | 2332 | + |
| Eselsburger Tal | I | *Polygala vulgaris* | 80 | + |
| Eselsburger Tal | II | *Asperula cynanchica* | 7715.4 | + |
| Eselsburger Tal | II | Asteraceae sp2 | 395 | + |
| Eselsburger Tal | II | *Campanula rotundifolia* | 83 | + |
| Eselsburger Tal | II | *Centaurea jacea* | 8 | + |
| Eselsburger Tal | II | *Cirsium arvense^+^* | 8 | - |
| Eselsburger Tal | II | *Dianthus carthusianorum** | 36 | - |
| Eselsburger Tal | II | *Echium vulgare* | 6 | + |
| Eselsburger Tal | II | *Galium verum* | 20160 | + |
| Eselsburger Tal | II | *Heliantemum nummularium* | 118 | + |
| Eselsburger Tal | II | *Lotus corniculatus* | 170 | + |
| Eselsburger Tal | II | *Ononis spinosa* | 818.4 | + |
| Eselsburger Tal | II | *Prunella vulgaris* | 122.4 | + |
| Eselsburger Tal | II | *Scabiosa columbaria* | 7 | + |
| Eselsburger Tal | II | *Sedum acre* | 39 | + |
| Eselsburger Tal | II | *Silene vulgaris** | 87 | - |
| Eselsburger Tal | II | *Teucrium chamaedrys* | 589 | + |
| Eselsburger Tal | II | *Teucrium montanum* | 793.6 | + |
| Eselsburger Tal | II | *Thymus pulegioides* | 2761.6 | + |
| Eselsburger Tal | II | *Trifolium dubium* | 706 | + |
| Eselsburger Tal | II | *Trifolium pratense* | 5 | + |
| Eselsburger Tal | III | *Aethusa cynapium* | 4004 | + |
| Eselsburger Tal | III | Asteraceae sp2*^+^* | 5 | - |
| Eselsburger Tal | III | *Campanula rotundifolia* | 7 | + |
| Eselsburger Tal | III | *Centaurea jacea* | 4 | + |
| Eselsburger Tal | III | *Cirsium arvense* | 9 | + |
| Eselsburger Tal | III | *Dianthus carthusianorum** | 1 | - |
| Eselsburger Tal | III | *Ononis spinosa* | 17.6 | + |
| Eselsburger Tal | III | *Scabiosa columbaria* | 5 | + |
| Eselsburger Tal | III | *Thymus pulegioides* | 128 | + |
| Hirschtal | I | *Ajuga reptans* | 28 | + |
| Hirschtal | I | *Cerastium arvense^+^* | 18 | - |
| Hirschtal | I | *Euphorbia cyparissias* | 1740 | + |
| Hirschtal | I | *Lotus corniculatus* | 70 | + |
| Hirschtal | I | *Polygala vulgaris^+^* | 20 | - |
| Hirschtal | I | *Ranunculus* sp. | 244 | + |
| Hirschtal | I | *Salvia pratensis* | 926 | + |
| Hirschtal | I | *Tripleurospermum inodorum* | 89 | + |
| Hirschtal | I | *Veronica chamaedrys* | 234 | + |
| Hirschtal | II | *Achillea millefolium^+^* | 57.6 | - |
| Hirschtal | II | *Agrimonia eupatoria* | 691.2 | + |
| Hirschtal | II | *Asperula cynanchica* | 5770.8 | + |
| Hirschtal | II | Asteraceae sp | 7 | + |
| Hirschtal | II | *Coronilla varia* | 79.2 | + |
| Hirschtal | II | *Dianthus carthusianorum** | 11 | - |
| Hirschtal | II | *Galium verum* | 1416 | + |
| Hirschtal | II | *Heliantemum nummularium* | 14 | + |
| Hirschtal | II | *Hypericum perforatum* | 9 | + |
| Hirschtal | II | *Lotus corniculatus* | 1032.4 | + |
| Hirschtal | II | *Origanum vulgare* | 154 | + |
| Hirschtal | II | *Salvia verticillata* | 120 | + |
| Hirschtal | II | *Scabiosa columbaria* | 133 | + |
| Hirschtal | II | *Stachys recta* | 216.6 | + |
| Hirschtal | II | *Teucrium chamaedrys* | 90 | + |
| Hirschtal | II | *Thymus pulegioides* | 698.6 | + |
| Hirschtal | II | *Trifolium campestre* | 240 | + |
| Hirschtal | II | *Trifolium pratense* | 21 | + |
| Hirschtal | II | *Tripleurospermum inodorum^+^* | 9 | - |
| Hirschtal | III | *Achillea millefolium* | 240 | + |
| Hirschtal | III | *Aethusa cynapium* | 2275 | + |
| Hirschtal | III | *Agrimonia eupatoria* | 684 | + |
| Hirschtal | III | *Asperula cynanchica* | 16650 | + |
| Hirschtal | III | *Cirsium arvense* | 9 | + |
| Hirschtal | III | *Clinopodium vulgare* | 156 | + |
| Hirschtal | III | *Daucus carota* | 2890 | + |
| Hirschtal | III | *Dianthus carthusianorum** | 16 | - |
| Hirschtal | III | *Galium verum* | 2760 | + |
| Hirschtal | III | *Heliantemum nummularium* | 2 | + |
| Hirschtal | III | *Hypericum perforatum* | 18 | + |
| Hirschtal | III | *Lotus corniculatus* | 98.6 | + |
| Hirschtal | III | *Origanum vulgare* | 5852 | + |
| Hirschtal | III | *Prunella vulgaris* | 9.6 | + |
| Hirschtal | III | *Scabiosa columbaria* | 270 | + |
| Hirschtal | III | *Teucrium chamaedrys^+^* | 60.8 | - |
| Hirschtal | III | *Thymus pulegioides* | 5760 | + |
| Hirschtal | III | *Trifolium dubium* | 65 | + |
| Hirschtal | III | *Trifolium pratense* | 4 | + |
| Reichenbach | I | *Euphorbia cyparissias* | 731 | + |
| Reichenbach | I | *Lotus corniculatus* | 183 | + |
| Reichenbach | I | *Onobrychis viciifolia* | 774 | + |
| Reichenbach | I | *Polygala vulgaris* | 200 | + |
| Reichenbach | I | *Ranunculus* sp*^+^* | 11 | - |
| Reichenbach | I | *Salvia pratensis* | 1358 | + |
| Reichenbach | I | *Trifolium repens* | 28 | + |
| Reichenbach | I | *Tripleurospermum inodorum* | 49 | + |
| Reichenbach | I | *Vincetoxicum hirundinaria* | 2940 | + |
| Reichenbach | II | *Agrimonia eupatoria* | 331.2 | + |
| Reichenbach | II | *Arnica montana** | 213 | - |
| Reichenbach | II | *Asperula cynanchica* | 16182 | + |
| Reichenbach | II | Asteraceae sp2 | 972 | + |
| Reichenbach | II | *Cichorium intibus* | 24 | + |
| Reichenbach | II | *Daucus carota^+^* | 4080 | - |
| Reichenbach | II | *Echium vulgare* | 96 | + |
| Reichenbach | II | *Galium verum* | 40800 | + |
| Reichenbach | II | *Heliantemum nummularium* | 41 | + |
| Reichenbach | II | *Leucanthemum vulgare* | 155 | + |
| Reichenbach | II | *Lotus corniculatus* | 183.6 | + |
| Reichenbach | II | *Medicago falcata* | 19 | + |
| Reichenbach | II | *Melampyrum arvense* | 85 | + |
| Reichenbach | II | *Ononis spinosa* | 510.4 | + |
| Reichenbach | II | *Origanum vulgare* | 66 | + |
| Reichenbach | II | *Prunella vulgaris* | 1603.2 | + |
| Reichenbach | II | *Scabiosa columbaria* | 24 | - |
| Reichenbach | II | *Senecio erucifolius* | 306 | + |
| Reichenbach | II | *Thymus pulegioides* | 1881 | - |
| Reichenbach | II | *Trifolium pratense* | 37 | + |
| Reichenbach | III | *Agrimonia eupatoria* | 136.8 | + |
| Reichenbach | III | *Anthemis tinctoria* | 193 | + |
| Reichenbach | III | *Asperula cynanchica* | 7812 | + |
| Reichenbach | III | Asteraceae sp2*^+^* | 811 | - |
| Reichenbach | III | *Aster amellus** | 6 | - |
| Reichenbach | III | *Campanula rotundifolia* | 239 | + |
| Reichenbach | III | *Centaurea jacea* | 6 | + |
| Reichenbach | III | *Cichorium intibus* | 41 | + |
| Reichenbach | III | *Cirsium arvense* | 25 | + |
| Reichenbach | III | *Coronilla varia* | 9 | + |
| Reichenbach | III | *Daucus carota* | 44370 | + |
| Reichenbach | III | *Galium verum* | 16920 | + |
| Reichenbach | III | *Heliantemum nummularium* | 10 | + |
| Reichenbach | III | *Leucanthemum vulgare* | 21 | + |
| Reichenbach | III | *Lotus corniculatus* | 782 | + |
| Reichenbach | III | *Medicago falcata* | 124 | + |
| Reichenbach | III | *Ononis spinosa* | 2037.2 | + |
| Reichenbach | III | *Origanum vulgare* | 1034 | + |
| Reichenbach | III | *Prunella vulgaris* | 1531.2 | + |
| Reichenbach | III | *Scabiosa columbaria* | 42 | + |
| Reichenbach | III | *Senecio erucifolius* | 210.8 | + |
| Reichenbach | III | *Teucrium chamaedrys* | 95 | + |
| Reichenbach | III | *Thymus pulegioides* | 1241.6 | + |
| Reichenbach | III | *Trifolium pratense* | 68 | + |

*protected species

^+^species with no fully wilted flowers or only one to four individuals flowering

*Additional file 1-7: Rarefaction curves for each community*

**
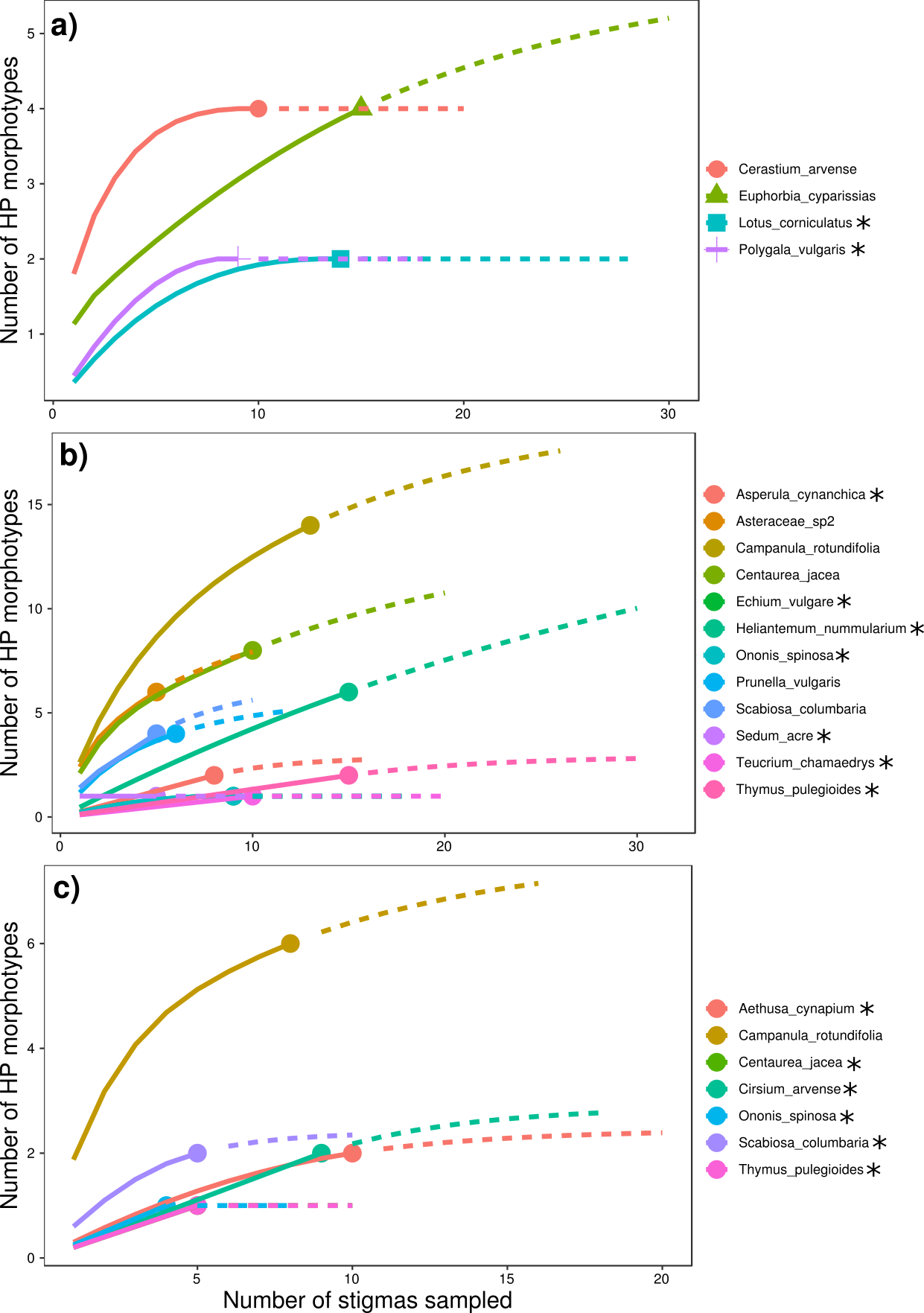
**

**Additional file 1-7.1**. Rarefaction and extrapolation curves Eselsburger Tal communities separated between early summer (a), mid summer (b) and late summer (c). Only species which received HP grains for at least one other species are shown. Confidence intervals are omitted for visibility. Asterisks behind plant species names indicate that data were insufficient to provide reliable estimators. Solid lines are interpolated results, dashed lines are extrapolated results.


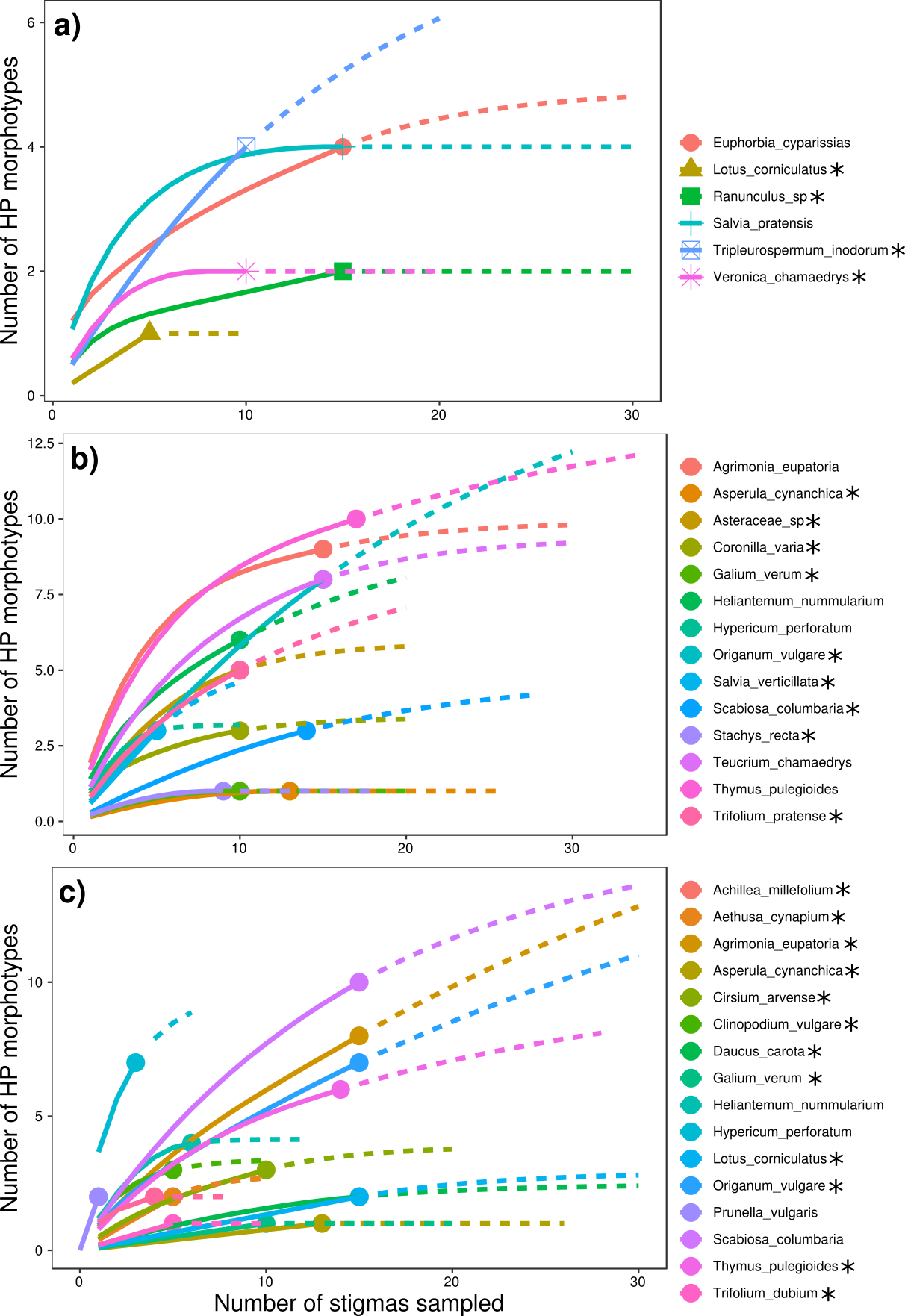


**Additional file 1-7.2**. Rarefaction and extrapolation curves Hirschtal communities separated between early summer (a), mid summer (b) and late summer (c). Only species which received HP grains for at least one other species are shown. Confidence intervals are omitted for visibility. Asterisks behind plant species names indicate that data were insufficient to provide reliable estimators. Solid lines are interpolated results, dashed lines are extrapolated results.


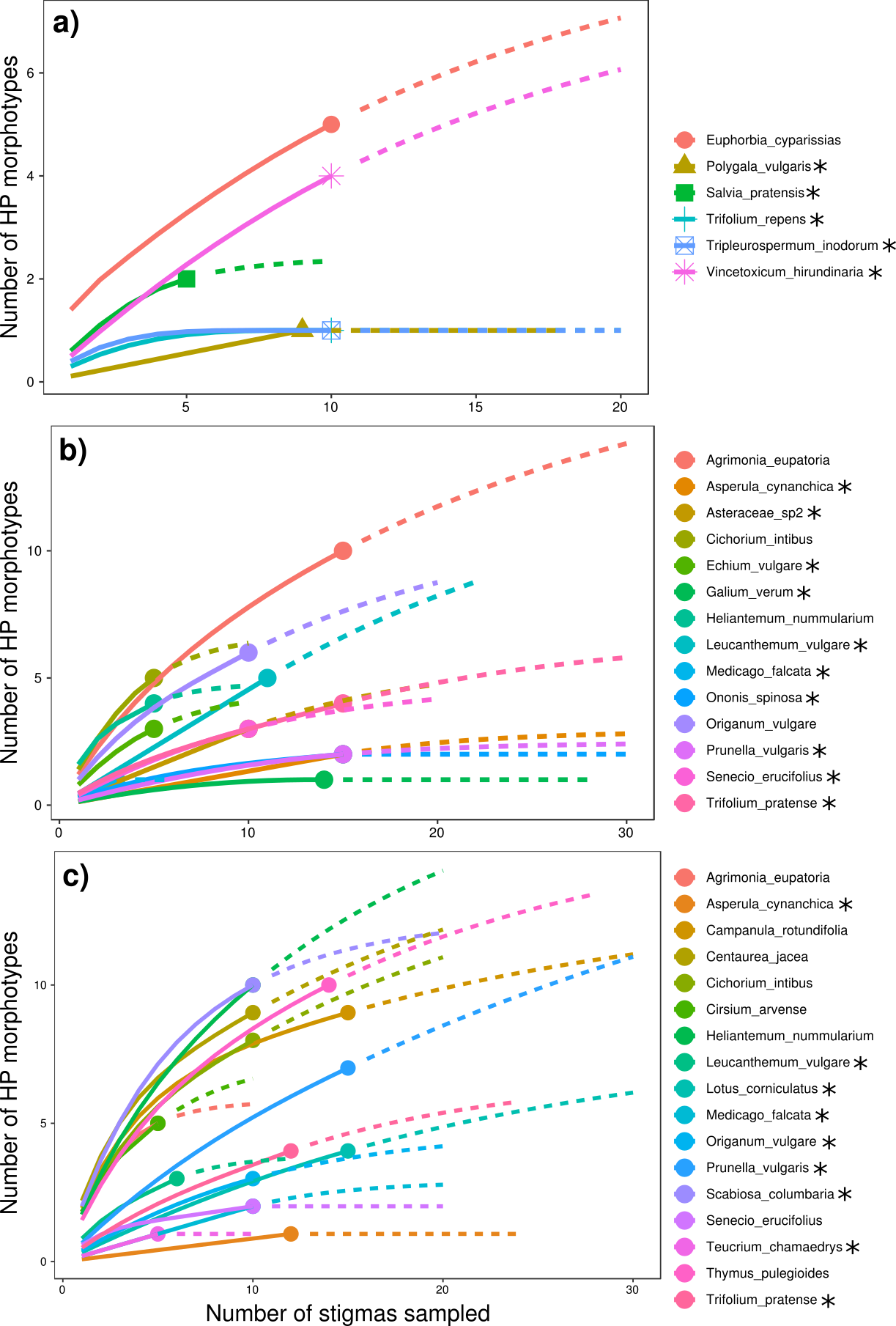


**Additional file 1-7.3**. Rarefaction and extrapolation curves Reichenbach communities separated between early summer (a), mid summer (b) and late summer (c). Only species which received HP grains for at least one other species are shown. Confidence intervals are omitted for visibility. Asterisks behind plant species names indicate that data were insufficient to provide reliable estimators. Solid lines are interpolated results, dashed lines are extrapolated results.

**References**

Bastian, M., Heymann, S. & Jacomy, M. (2009). *Gephi: an open source software for exploring and manipulating networks*. *Int. AAAI Conf. Weblogs Soc. Media*. Available at: www.gephi.org. Last accessed 6 January 2019.

Blondel, V.D., Guillaume, J.L., Lambiotte, R. & Lefebvre, E. (2008). Fast unfolding of communities in large networks. *J. Stat. Mech. Theory Exp.*, 2008.

Cribari-Neto, F. & Zeileis, A. (2010). Beta regression in R. *J. Stat. Softw.*, 34, 1–24.
